# Supplementary material for: HIVprotI: an integrated web based platform for prediction and design of HIV proteins inhibitors
Source: J Cheminform. 2018 Mar 9;10:12. doi: 10.1186/s13321-018-0266-y (PMC5845081; doi:10.1186/s13321-018-0266-y)
Supplement: Supplementary file 2 — Additional file 2. Source code of HIVProtI web server. [file 13321_2018_266_MOESM2_ESM.zip › HIVprotI_Source-code/hivprotI/jsme/JSME_hidden.html]

# JSME in a hidden div

## The size is fixed (defined in px)

Show
Hide
  
  


---

  
  


---

## The size is dynamic (defined in %)

Note: jsmeApplet2.repaint() must be called after displaying.
  
Show
Hide
  
  


---
